# The HIV Anxiety Scale (HAS): Developing and Validating a Measure of Human Immunodeficiency Virus (HIV) Anxiety

**DOI:** 10.1007/s10461-025-04690-2

**Published:** 2025-04-05

**Authors:** Liam Cahill, Anthony J. Gifford, Bethany A. Jones, Daragh T. McDermott

**Affiliations:** 1 NTU Psychology, School of Social Sciences, Nottingham Trent University, 50 Shakespeare Street, Nottingham, NG1 4FQ England, UK; 2 College of Psychology, Birmingham City University, BLSS, Birmingham, UK

**Keywords:** HIV anxiety, HIV, Psychometric measure, Prevention

## Abstract

Most research assessing human immunodeficiency virus (HIV) anxiety relies on single-item measures or psychometric measures that are outdated in terms of concepts and language. There is a critical need for a robust, reliable, and contemporary measure to identify populations at risk of avoiding HIV testing, treatment, and prevention, thereby supporting global HIV eradication goals. Focus groups informed the initial development of the HIV Anxiety Scale (HAS), revised through expert feedback. The factor structure was assessed in two studies. In Study 1, an Exploratory Factor Analysis (EFA) was conducted with 251 participants. In Study 2, a Confirmatory Factor Analysis (CFA) with 200 participants was performed alongside validity, internal consistency, and measurement invariance assessments. Studies 1 and 2 elicited a 3-factor model, resulting in a 16-item measure with the following subscales: *Psychosocial Implications of HIV*,* Lifestyle Implications of HIV*,* and HIV Testing Anxiety*. The HAS demonstrated a good factor structure, acceptable validity and excellent internal consistency across diverse groups in Study 2. The HAS provides a contemporary, robust measure of HIV anxiety, addressing limitations of previous tools and contributing to efforts to identify and support populations at risk of HIV avoidance behaviours. We recommend that future research continue to validate and test this new measure, but it offers a standardised tool to inform targeted interventions for HIV testing, prevention, and treatment.

## Resumen

La mayoría de las investigaciones que evalúan la ansiedad relacionada con el VIH se basan en medidas de un solo ítem o en herramientas psicométricas desactualizadas en términos de conceptos y lenguaje. Existe una necesidad crítica de contar con una medida sólida, confiable y contemporánea para identificar a las poblaciones en riesgo de evitar las pruebas, el tratamiento y la prevención del VIH, apoyando así los objetivos globales de erradicación del VIH. Los grupos de enfoque informaron el desarrollo inicial de la* HIV Anxiety Scale (HAS)*, revisada a través de comentarios de expertos. La estructura factorial fue evaluada en dos estudios. En el Estudio 1, se realizó un Exploratory Factor Analysis (EFA) con 251 participantes. En el Estudio 2, se llevó a cabo un Confirmatory Factor Analysis (CFA) con 200 participantes, junto con evaluaciones de validez, consistencia interna e invarianza de la medición. Los estudios 1 y 2 revelaron un modelo de 3 factores, dando lugar a una medida de 16 ítems con las siguientes subescalas: Implicaciones Psicosociales del VIH, Implicaciones del VIH en el Estilo de Vida y Ansiedad por la Prueba del VIH. La HAS demostró una buena estructura factorial, validez aceptable y excelente consistencia interna en diversos grupos en el Estudio 2. La HAS proporciona una medida contemporánea y robusta de la ansiedad por HIV, abordando las limitaciones de herramientas anteriores y contribuyendo a los esfuerzos para identificar y apoyar a las poblaciones en riesgo de conductas de evitación relacionadas con el VIH. Recomendamos que futuras investigaciones continúen validando y probando esta nueva medida, pero ofrece una herramienta estandarizada para informar intervenciones específicas en pruebas, prevención y tratamiento del VIH.

## Introduction

Human immunodeficiency virus (HIV) is a chronic viral infection that impacts approximately 39.0 million people worldwide [1]. The Joint United Nations Programme on HIV/AIDS (UNAIDS) has set ambitious targets to eradicate new transmissions of HIV worldwide by 2030, with widespread testing, treatment, and pre-exposure prophylaxis (PrEP) being key focuses [2]. Despite this global effort, in the United Kingdom (UK), recent data indicate a 22% increase in new diagnoses, with gay and bisexual men who have sex with men and heterosexual women being most disproportionately affected [3]. The persistence of transmission disparities, particularly among these groups, highlights the need for targeted interventions addressing psychosocial processes, including stigma and anxiety surrounding HIV testing and treatment, which remain significant barriers to effective prevention and treatment strategies [4]. These issues must be systematically addressed if the UK wishes to achieve ambitious national targets for eradicating HIV transmission and ensuring that those who are seropositive for HIV are on effective treatment.

### The Impact of HIV Anxiety

The understanding that those living with HIV who are on effective antiretroviral therapy resulting in an undetectable viral load are unable to transmit the virus is a breakthrough in reducing stigma and anxiety around HIV [5]. Yet, the challenges in communicating the *undetectable equals untransmissible* (U = U) health messaging (e.g., due to scepticism and stigma) show the need for further work in reducing unequal access to HIV prevention and treatment, as well as wider societal stigmas [6]. The stigmatisation of HIV often results in increased anxiety around the virus, especially amongst minoritised populations such as gay and bisexual men who have sex with men [7]. A substantial body of research highlights how stigma and fear inhibit open discussions of safer sex practises, discourage testing and treatment, and perpetuate poor treatment adherence, all increasing the risk of HIV transmission [8, 9]. Stigma and fear of HIV are, therefore, intertwined with anxiety around acquiring the virus (i.e., HIV anxiety), which acts as a major barrier to reducing HIV infections [10]. Here, we define HIV anxiety as the apprehension, fear, or worry about contracting or being diagnosed with HIV, often shaped by stigma, misinformation or lack of knowledge, and perceived risk of infection [11–13].

HIV anxiety can have several detrimental effects on health outcomes [14]. One significant consequence of late HIV diagnosis is a ninefold increase in risk of progression to acquired immunodeficiency syndrome (AIDS) and mortality, accompanied by elevated healthcare costs, a greater burden on healthcare systems, and a substantial reduction in the quality of life for affected individuals [4]. In 2021, 43% of HIV diagnoses in the UK were classified as late, underscoring the critical need for earlier detection and intervention to mitigate severe health outcomes [15]. Currently, we know very little about the factors that influence HIV anxiety and ways to prevent it.

The psychological stressors of perception of HIV risk and HIV-related anxieties have also been cited as a major barrier to engaging with HIV prevention services (see [16]). Findings indicated that greater misconceptions about HIV infectiousness (i.e., believing that HIV medication does not prevent transmission), and lower treatment optimism (i.e., limited belief in the efficacy of HIV medication) were associated with greater tendencies to avoid HIV information, which, in turn, were linked to never having been tested or not being tested recently. The avoidance of HIV information can act as a proximal barrier to HIV testing, as well as diminish awareness of other HIV prevention modalities, such as PrEP [17]. PrEP is among the most effective methods of reducing HIV transmission rates but is often underutilised, especially among the populations most at risk of HIV acquisition [18, 19]. Indeed, PrEP uptake is not merely impacted by the perceived risk of HIV alone but by psychological determinants such as stigma and health beliefs as well [20]. Broader research also indicates how access to HIV prevention (e.g., PrEP) can help alleviate HIV-related anxieties [21]. This has added benefits such as improving mental health and quality of life outcomes for those at risk of increased marginalisation (e.g., gay and bisexual men who have sex with men). There are also calls to include HIV-related anxieties as a factor when assessing clinical candidacy for PrEP [22]. Therefore, understanding HIV anxiety as a psychological concept has major implications for improving the quality of life and health outcomes of those impacted by the virus.

However, despite a broad examination of HIV anxiety across many related disciplines (e.g., psychology, sociology, public health, and epidemiology), there is no universal operationalised measure of HIV-related anxieties. Many studies use single-measure items to measure the extent of HIV worry (e.g [16, 20]), or non-specific anxiety-related measures (e.g., Brief Symptom Inventory; [23]). Other related measures are also outdated and use language that is now deemed inappropriate (e.g., The multidimensional AIDS anxiety questionnaire: ‘*I am afraid of contracting AIDS through casual contact with others*’; [24]). Such language misrepresents the biological mechanisms of HIV transmission and reinforces the misconception that AIDS itself is an infectious condition [25]. In the era of *U = U* and widely available prevention tools like PrEP, outdated or generalised measures of HIV anxiety likely fail to capture the nuances of HIV anxiety for HIV-negative individuals. Finally, much research also explores anxiety-related symptoms in those living with HIV [26, 27]. There is a growing need to address the anxiety experienced by HIV-negative individuals, particularly in the context of evolving prevention strategies, as many studies have primarily focused on mental health outcomes within HIV-positive populations [28, 29], overlooking the unique concerns of those at risk for HIV. There is a distinct need to establish an understanding of HIV anxiety in a contemporary context where HIV is both manageable and preventable.

### The Present Research

There is currently a lack of validated, contemporary measures of HIV anxiety. As such, a measure is needed, given that HIV anxiety in HIV-negative people may partly explain why these individuals do not undergo regular or systematic HIV testing [30]. Similarly, HIV anxiety, like other health anxieties, has implications for an individual’s health and well-being and quality of life [7]. Thus, a validated measure of HIV anxiety is warranted and could be utilised both in healthcare settings (e.g., to identify individuals most likely to avoid testing) and by researchers.

The current research aims to develop and validate the HIV Anxiety Scale (HAS) in a general sample. Since we intend for this measure to be used widely, we aim to recruit a relatively diverse population from the UK. That is, while some groups, such as men who have sex with men, are generally more at risk of HIV, we aim for this initial validation of the HAS to be with a general sample, allowing future research to validate this measure with specific at-risk groups. To achieve this, we will complete two studies.

Study 1: We will create the initial HIV Anxiety Scale (HAS) item pool and refine it by gathering feedback from experts in the field and participants during focus groups. We will then conduct exploratory factor analysis (EFA) to determine the best factor structure for the proposed measure.

Study 2: We will perform confirmatory factor analysis (CFA) on the measure developed in Study 1 and assess its construct validity and internal consistency. For this study, we make the following pre-registered hypotheses:

#### H1:

We hypothesise that all items will significantly load onto their respective factors and that the confirmatory factor analysis (CFA) will show good model fit, as evidenced by examining the model fit indices (RMSEA, TLI, CFI, and SRMR).

#### H2:

We hypothesise that HIV anxiety, both as an overall measure and each subscale (*Psychosocial Implications of HIV*,* Lifestyle Implications of HIV*,* and HIV Testing Anxiety*), will be (1) positively correlated with health anxiety, (2) negatively correlated with quality of life and (3) positively correlated with perceived risk of HIV.

#### H3:

Given that HIV anxiety is conceptually distinct from the following constructs, we hypothesise that HIV anxiety, both as an overall measure and each subscale (*Psychosocial Implications of HIV*,* Lifestyle Implications of HIV*,* and HIV Testing Anxiety*), will (1) show no significant correlation with internet gaming disorder or (2) with social desirability.

## Study 1

### Method

#### Phase 1: Developing the HAS

The development of the HAS item pool comprised two stages. First, we created a broad pool of items tapping into various constructs (e.g., HIV testing anxiety). We consulted experts in HIV research, both academic and clinical, to gather informal feedback on a set of 53 items. Through iterative discussions with the research team and these specialists, we applied the following criteria to refine the item pool: (1) Removed duplicate items, retaining some duplicates to evaluate which wording worked best, (2) Excluded items with high conceptual overlap and (3) Eliminated items that were difficult to read or contained multiple clauses. (4) Consistent with the broader literature, it was recommended to employ the term ‘worry about’ as synonymous with ‘anxious about’ to underscore the cognitive component of anxiety rather than emphasising autonomic arousal [31]. Based on this process, we refined, added, and removed items as necessary, resulting in 39 items for use in focus groups. This initial pool is available on OSF.

Second, LC and AG conducted two focus groups comprising five and four participants. These participants identified as men who have sex with men (MSM). We sampled this participant group as MSM are more knowledgeable about HIV compared to the general population, reporting a greater understanding of HIV prevention methods, treatment options, and testing strategies, particularly in regions with extensive HIV prevention programs​ [14]. Furthermore, MSM generally experience higher levels of anxiety related to HIV compared to non-MSM populations [32]. This heightened awareness and lived experience with HIV make MSM an ideal population for evaluating the clarity and relevance of items intended to measure HIV anxiety. Since the primary aim of these focus groups was to assess whether the items effectively addressed HIV anxiety, we chose to sample a more informed and engaged audience to ensure their feedback was both nuanced and reflective of diverse experiences within this domain. This contrasts with phase two and study 2, where we aimed to explore and validate the actual HAS measure with a general sample.

During the focus groups, participants suggested various revisions to the measure. While we cannot list every change here, a detailed account is available on the Open Science Framework (OSF; https://osf.io/uwspe). Key recommendations were: (1) Participants noted confusion between four items; in response, we reworded two and removed the two others to enhance clarity. (2) They suggested eliminating certain items, leading us to remove four based on their feedback. (3) They highlighted the need to rephrase two items for better understanding. We reworded one item and split one item into two new ones. (4) Finally, they recommended simplifying the overall measure, which we addressed by adjusting the reading level to 9–12 years, ensuring consistency in the adjectives used for each item and changing the measure’s instructions to be more neutral. This resulted in a more refined 34-item HAS measure, which we used in Phase 2.

#### Phase 2: Exploratory Factor Analysis

***Participants and Design.*** In phase two, we administered the refined measure. We sampled 285 participants using Prolific, and participants were compensated 60p (£9/hr) for their time. The study took approximately 5 min to complete. We recruited participants using a quota-based sampling approach to ensure a diverse representation across key demographic variables. Quotas were applied for ethnicity, sexual orientation, and gender. This approach facilitated the recruitment of a sample encompassing a wide range of identities and experiences.

Our inclusion criteria required participants to be based in the UK, single, in mutually non-monogamous open relationships, or engaged in romantic and/or sexual relationships with multiple partners. We used this latter inclusion criteria as we believed participants with this relationship status would be more likely to be concerned about HIV and other sexual health issues. We also restricted our sample to participants who were either HIV-negative or did not know their status, as we believed these participants would be most concerned about HIV transmission. As the measure was presented in English, we restricted our sample to only those who spoke English as a first language.

Our final sample comprised 251 participants aged between 18 and 74 (*M*_age_ = 30.26, *SD*_age_ = 10.49). We manually removed one participant as they did not meet our HIV status inclusion criteria (i.e., they reported being HIV positive). We removed another as they failed two or more attention checks embedded in the survey. Finally, 32 participants were automatically screened out as they did not meet our other inclusion criteria (e.g., were not single). Table 1 presents the demographic information for our sample in studies one and two. Most of our participants (98%) reported being single, with the remaining 2% identifying as being in a relationship but seeing other people romantically or sexually or being in a mutually non-monogamous relationship. After answering the demographic questions, the participants then completed the HAS. The items were presented in randomised order.

**Table 1:** Demographic information for study one and two

Demographic question	Study one (*N* = 251)	Study two (*N* = 200)
	*n* (%)	*n* (%)
*What is the sex you were assigned at birth?*		
Male	123 (49)	85 (43)
Female	128 (51)	114 (57)
Prefer not to say	–	1
*How do you identify yourself in relation to your gender?*		
Woman	122 (48)	108 (54)
Man	114 (45)	83 (42)
Non-binary	9 (4)	3 (2)
Trans-woman	1	–
Bi-gender	1	1
Agender	1	–
No gender	1	–
Biological female	–	1
Genderfluid	–	1
Non-binary man	–	1
Other	1	1
*Is your current gender identity different to your sex assigned at birth?*		
Yes	105 (42)	75 (38)
No	145 (58)	123 (62)
Prefer not to say	1	2 (1)
*What is your HIV status?*		
I am HIV negative	199 (79)	170 (85)
I do not know my status	52 (21)	30 (15)
*How would you define your sexual orientation?*		
Straight	107 (43)	89 (45)
Bisexual	63 (25)	47 (24)
Gay	57 (23)	41 (21)
Pansexual	16 (6)	12 (6)
Queer	4 (2)	2 (1)
Prefer not to say	3 (1)	3 (2)
Demisexual/ Grey ACE	–	1
*How do you identify yourself in relation to your ethnicity?*		
White	125 (50)	120 (60)
Asian or Asian British	47 (19)	26 (13)
Mixed or multiple ethnic groups	40 (16)	27 (14)
Black, African, Caribbean or Black British	36 (14)	27 (14)
Other	2 (1)	–
Prefer not to say	1	–

The question “*How do you identify yourself in relation to your gender*?” was posed as a free-text question. The authors coded and manually categorised these responses. We preserved the participants’ original wording. However, for simplicity, we represented sex-based responses (e.g., “male” or “female”) as their gender-equivalent categories (i.e., “man” or “woman”). Participants who selected “other” for the sexual orientation question mostly specified “queer” in the free-text box. One participant for study two stated demisexual or Grey ACE (reflected above). Given the small frequencies, we did not present proportions for any individual categories (i.e., when only one participant chose that option). Proportions may not equal 100% due to rounding

***Statistical Analyses.*** Our analysis was conducted in R. The anonymised data that supports our analyses is available on the OSF. Since we assumed the factors we identified would be correlated, we used an oblique rotation method (i.e., Promax). Our analytical process occurred in several stages.

First, we employed multiple approaches to determine the appropriate number of factors to retain. We examined both (1) eigenvalues and (2) the scree plot. However, relying solely on these methods has several limitations. Therefore, we also applied (3) parallel analysis and (4) the minimum average partial (MAP) test, which are considered more precise [33]. While the MAP test was originally designed for use with principal component analysis, it is also a valid method of identifying factor structures within EFAs [34]. Our final decision on factor retention was guided by a comprehensive assessment of all these methods. Second, we explored the factor loadings after repeating the above steps. We retained only items that loaded onto a single factor by 0.50 or greater and had no cross-loadings exceeding 0.32 [35, 36]. Finally, we explored whether any of our items were redundant. That is, we investigated the inter-item correlations to determine if any of our items measured the same construct. If these correlations were equal to or greater than 0.70 (or − 0.70), we considered whether these should be removed. We examined these items and determined whether removing them made theoretical sense. If we did remove an item, we retained the item that loaded the highest onto the respective factor.

### Results

#### Preliminary Analyses

We identified a small number of missing responses (*n* = 3) on the HAS. We decided to impute these values using the multiple imputation for chained equations (MICE) package to ensure valid responses were not lost. We then assessed the appropriateness of our data for exploratory factor analysis (EFA). After removing the redundant items (see below), we determined that the assumptions of multicollinearity and sphericity were not violated. We checked whether our items had good common variance using the Kaiser–Meyer–Olkin (KMO) measure. We found that our items generally showed good common variance (0.93−0.98). However, item 30 (“*I feel relieved knowing effective HIV treatments are available*”) and item 31 (“*I feel hopeful about the advancements in HIV research and treatment*”) showed poor KMO scores of 0.66 and 0.63 respectively. As these items related to HIV treatment and were the only items relating to this concept in the measure, they likely measured a somewhat different concept relative to the other items. For this reason, we decided to delete these items before proceeding. Following this, we proceeded with our EFA as planned with the remaining 32 items.

#### Primary Analyses

Our primary analysis was an iterative process. We continuously assessed the inter-item correlations and factor loadings to determine the best factor solution. Table 2 displays the final factor solution. The eigenvalues, parallel analysis, and MAP test revealed that a three-factor structure was the most appropriate. However, the scree plot suggested two factors were the most appropriate. Given this, we proceeded with a three-factor solution.

**Table 2:** Factor loadings, proportion of explained variance and McDonald’s Omega for the final 16-item measure and the removed items

Item	Factor 1	Factor 2	Factor 3
*Factor 1: psychosocial implications of HIV*			
3 I worry that HIV will impact current or new intimate and romantic connections	**0.74**	− 0.10	0.04
4 I worry about the impact of HIV on my sex life	**0.73**	− 0.13	0.17
5 I worry about how living with HIV would affect my friendships	**0.77**	0.19	− 0.16
6 I am afraid my family would treat me differently if I were living with HIV	**0.63**	0.09	0.03
9 I fear people would judge me if I had HIV	**0.95**	− 0.06	− 0.06
14 I worry about how living with HIV would affect how I see myself	**0.63**	0.11	0.14
19 I worry that I would face discrimination at work if I had HIV	**0.51**	0.29	− 0.01
20 I fear I wouldn’t have anyone to talk to if I had HIV	**0.54**	0.14	0.01
*Factor 2: lifestyle implications of HIV*			
2 I worry about how living with HIV would affect my finances (i.e., money)	0.20	**0.53**	–
11 I worry that living with HIV will affect my career	0.17	**0.60**	0.12
12 I worry about my ability to travel if I have HIV	− 0.03	**0.70**	0.10
13 I worry that having HIV would affect my access to healthcare	0.14	**0.63**	− 0.08
15 I fear having HIV would affect my access to housing	− 0.19	**0.87**	0.03
*Factor 3: HIV testing anxiety*			
26 I’ve felt panicked when thinking about testing positive for HIV	0.26	− 0.08	**0.65**
27 I’ve felt panicked when thinking about having an HIV test	− 0.20	0.12	**0.97**
28 The idea of waiting for HIV test results makes me feel overwhelmed	0.22	0.03	**0.60**
Variance explained (%)	29	18	13
McDonald’s Omega (ω)	0.91 [0.89, 0.92]	0.83 [0.79, 0.87]	0.86 [0.81, 0.88]
Mean (standard deviation)	250.24 (80.79)	90.48 (40.33)	80.31 (30.72)
*Removed items*			
1 I worry about acquiring HIV and the impact this would have on my life^Cross^	0.44	0.01	0.38
7 I worry that having HIV would make me feel isolated^Inter−Item^	–	–	–
8 I worry that people will see me differently if I have HIV^Inter−Item^	–	–	–
10 I worry about how people in my community would treat me if I had HIV^Inter−Item^	–	–	–
16 I fear having HIV would significantly impact my daily routine^Loading^	0.31	0.35	0.21
17 I worry that having HIV will affect my leisure activities^Loading^	0.31	0.49	0.03
18 I fear the long-term effects of taking HIV medication^Loading^	0.24	0.38	0.13
21 I am uneasy about how the media affects public views and the stigma around HIV^Loading^	0.44	0.17	< 0.01
22 My heart beats faster when I think about testing positive for HIV^Inter−Item^	–	–	–
23 My heart beats faster when I think about having an HIV test^Inter−Item^	–	–	–
24 I have a sense of dread when thinking about testing positive for HIV^Cross^	0.45	− 0.07	0.49
25 I have a sense of dread when thinking about having an HIV test^Inter−Item^	–	–	–
29 I feel ashamed when I think about possibly having HIV^Cross^	0.39	0.13	0.32
32 I feel upset when I think about how my life might change because of HIV^Cross^	0.47	0.11	0.33
33 I would rather not know I had HIV until I needed to^Loading^	− 0.24	0.4	0.39
34 I would do anything to avoid acquiring HIV^Loading^	0.43	− 0.05	0.06

Salient factors are in bold. The reason for item removal is shown in superscript: *Cross* = cross loading, *Loading* = did not load onto a factor, and *Inter-Item* = item correlated highly with another item. Items 30 and 31 were removed during the initial stages before conducting the EFA. Values contained within square brackets represent the 95% Confidence Interval

We identified several high inter-item correlations (i.e., ≥ 0.70). We expected this given the built-in redundancy of the initial item pool. Items 7, 8, 9 and 10 were all correlated highly with each other. This is expected as each of these items taps into different elements of the social experience around HIV anxiety. Based on the factor loadings, we decided to delete items 7, 8 and 10 and retain item 9. Other items also showed relatively high correlations, including between items 22, 23, 25, 26, 27 and 28. Again, this is expected given that these items measure anxiety around HIV testing. Based on the factor loadings, we deleted items 22, 23 and 25. While items 26 and 27 were correlated (0.70), we decided to retain these because it was logical that an item relating to panic relating to HIV testing would be associated with an item related to panicking about a positive test result. Despite the correlation, each of these items measures a distinct aspect of HIV anxiety. As such, we removed six items at this stage and proceeded with the EFA on the remaining 26 items.

We next assessed the factor loadings for the remaining items. We removed six items as they failed to load sufficiently onto any single factor. We identified four additional items that cross-loaded onto two or more factors. They loaded onto two or more factors with a loading of 0.32 or greater. These items also did not load sufficiently onto any one factor. Item 1 (“*I worry about acquiring HIV and the impact this would have on my life*”) loaded onto factors one (*Psychosocial Implications of HIV*) and three (*HIV Testing Anxiety*). This cross-loading is likely due to this question tapping into both the psychosocial concerns associated with HIV and the anxieties around testing. However, as this item did not load sufficiently onto either factor, we removed it.

Item 24 (“*I have a sense of dread when thinking about testing positive for HIV*”) loaded almost equally onto factors 1 and 3. Logically, this item would be associated with both factors, given the potential psychosocial impacts associated with testing positive, alongside the anxieties around HIV testing. Given the high cross-loading and the item not loading sufficiently onto either factor, we decided to remove this.

Item 29 (“*I feel ashamed when I think about possibly having HIV*”) cross-loaded onto factors one and three. The shame when considering the possibility of having HIV may influence both their psychosocial processes (e.g., interpersonal relationships) and their HIV testing anxiety, given that people may fear judgment from healthcare professionals for having an HIV test or for potentially receiving a positive test result. Given the high cross-loading and the item not loading sufficiently onto either factor, we decided to remove this.

Finally, Item 32 (“*I feel upset when I think about how my life might change because of HIV*”) cross-loaded onto factors one and three. This is likely because the distress associated with potential life changes due to HIV affects both domains. That is, the item reflects broader implications for how living with HIV would influence psychosocial processes. Similarly, the anxiety about these potential life changes can also contribute to heightened emotional responses when considering HIV testing, as the outcome of the test could trigger those feared changes. Given the high cross-loading and the item not loading sufficiently onto either factor, we decided to remove this.

These steps resulted in a 16-item measure (Table 2) comprising three subscales. The first subscale reflected the *Psychosocial Implications of HIV* (*n* = 8), the second reflected the *Lifestyle Implications of HIV* (*n* = 5), and the third reflected *HIV Testing Anxiety* (*n* = 3). The internal consistency of each subscale (Table 2) was good, similar to the overall measure’s internal consistency (ω = 0.94, 95% CI: 0.93, 0.95). Table 2 also presents the descriptive statistics (means and standard deviation) for each subscale (overall measure: *M* = 42.75, *SD* = 14.94).

## Study 2

We pre-registered the hypotheses and materials for this study on the OSF: https://osf.io/b872c.

### Method

#### Participants and Design

We used the same inclusion criteria and sampling approach (i.e., quota sampling) as Study 1. Our final sample comprised 200 participants (*M*_age_ = 31.31, *SD*_age_ = 10.85). We manually removed three participants as they did not meet our HIV status inclusion criteria (i.e., they indicated that they preferred not to indicate their HIV status). Nineteen participants were automatically screened out as they did not meet our inclusion criteria. We removed an additional participant as they failed two or more attention checks embedded within the study. Most (98.5%) of the participants were single, with the remaining participants identifying as being in a relationship but seeing other people romantically or sexually or being in a mutually non-monogamous relationship. Table 1 presents the demographic information for Study 2’s sample.

An a-priori sensitivity power analysis using the SemPower package in R indicated 200 participants were sufficient to correctly identify a model with a misspecification of RMSEA = 0.05, an alpha of 0.05 and a power of 0.80. Using the pwr package in R, this sample was also sufficient to detect a weak correlation (*r* = 0.20).

#### Measures

After completing the demographic questions in Table 1, the participants completed the following measures. The order of the measures and the order of the questions within each measure was randomised.

***HIV Anxiety Scale (HAS).*** We administered the 16-item HAS developed in Study 1. As mentioned, this measure comprises three subscales: Psychosocial Implications of HIV, Lifestyle Implications of HIV and HIV Testing Anxiety. Participants responded to each of the 16 items on a 5-point rating scale, ranging from *not at all* (1) to *very much* (5). The specific anchor points used were (1) not at all, (2) a little, (3) somewhat, (4) quite a bit and (5) very much. The participants were asked to indicate how much each statement reflects their thoughts and feelings. Both total scores and scores on each of the measure’s subscales were calculated by computing a mean score for each participant. Higher scores reflected greater HIV anxiety, both overall and for each subscale.

***World Health Organisation Quality of Life Scale (WHOQoL BREF;*** [37]***).*** The WHOQoL BREF scale is a 24-item measure of a person’s quality of life. The scale measures a person’s quality of life across four domains: psychological (6 items; “*How much do you enjoy life?*”), physical (7 items; “*How satisfied are you with your sleep?*”), social (3 items; “*How satisfied are you with your sex life*?”) and environmental quality of life (8 items; “*How safe do you feel in your daily life?*”). We calculated a mean score across these four domains for each participant to compute an overall quality of life score. As such, higher overall scores indicated greater quality of life. This measure was used to assess convergent validity.

***Short Health Anxiety Inventory (S-HAI;*** [38]***).*** The S-HAI is a cluster of 14 items, with each cluster containing four statements. The 14-item version is the shortest version of the S-HAI. Participants responded to each question by indicating the option that best described their feelings over the last 6 months. For example, participants indicate which of the following best describes them: (a) “*I do not worry about my health”*, (b) “*I occasionally worry about my health*”, (c) “*I spend much of my time worrying about my health*” or (d) “*I spend most of my time worrying about my health*”. A total score was calculated by summing across each of the 14 items. Higher scores indicated greater levels of health anxiety. This measure was used to assess convergent validity.

***Perceived Risk of HIV Scale (PRHS;*** [12]***).*** The PRHS has 8 items which measure the extent to which a person believes they are at risk of HIV (e.g., “*What is your gut feeling about how likely you are to get infected with HIV?*). The measure consists of different response options for each item (e.g., *extremely unlikely* to *extremely likely* and *none of the time* to *all of the time*). The rating scale also changes depending on the item, with some items responded to on a four-, five- or six-point rating scale. Total scores were calculated for each participant. Higher scores indicated a greater perceived risk of HIV. This measure was used to assess convergent validity.

***Internet Gaming Disorder Scale– Short Form (IGDS;*** [39]***).*** The IGDS comprises a 9-item measure of internet gaming disorder. Participants respond based on their gaming activity over the past year. Gaming is defined as any gaming-related activity played from a computer/, laptop gaming console, or any other device. Participants respond to each item (e.g., “*Do you feel more irritability*,* anxiety or even sadness when you try to either reduce or stop your gaming activity?*”) on a five-point scale ranging from *strongly disagree* (1) to *strongly agree* (5). A total score was created by summing the participant’s responses. Higher scores indicated higher IGD. This measure was used to assess divergent validity. We decided on this measure as we believed it to be conceptually unrelated to HIV anxiety. Previous measure development papers have also used this measure for this purpose [see: 40].

***Marlowe-Crowne Social Desirability Scale (M-C SDS;*** [41]***).*** The M-C SDS is a measure of socially desirable responding. We used the 13-item short-form version of the scale Reynolds (1982) developed to reduce participant burden. Participants respond to each of the 13 items (e.g., “*I have never deliberately said something to hurt someone’s feelings*”) on a binary response scale (*True* or *False*). A social desirability score can be obtained by summing each socially desirable response. As such, higher scores indicate more socially desirable responses. This measure was used to assess divergent validity.

#### Statistical Analyses

The data and R code that support our following analysis can be found on the OSF. We first examined the normality of the individual HAS items and each total and subscale score for each of our measures. We determined that the univariate normality assumption was not violated if the skew and kurtosis did not exceed ± 2 [42].

We then conducted a confirmatory factor analysis using the *lavaan* package in R. We used (due to no violations to normality, see below) a maximum likelihood estimator when fitting our CFA. We evaluated model fit using several key indices to explore our model fit: the Root Mean Square Error of Approximation (RMSEA), Tucker-Lewis Index (TLI), Comparative Fit Index (CFI), and Standardised Root Mean Square Residual (SRMR). Common guidelines suggest that a model demonstrates a good fit when CFI and TLI values are 0.95 or higher, RMSEA is 0.06 or below, and SRMR is 0.08 or lower. Similarly, a CFI and TLI between 0.90 and 0.94, RMSEA between 0.07 and 0.10, and SRMR between 0.09 and 0.10 indicates an acceptable model fit [43]. Given the issues with using the chi-square test to determine model fit, which is sensitive to model complexity and sample size, we will report this for clarity but not to inform our decisions around model fit [44].

We then explored whether the 16 items within the HAS loaded sufficiently onto their respective factors. Consistent with common practice, we used a relatively conservative cut-off point of 0.50 to determine whether our items load sufficiently onto the respective factors [45].

To assess the construct validity of the HAS measure, we computed correlations (*r*) between the HAS total and subscale scores and the other measures to assess convergent and divergent validity. We also computed the internal consistency for the final measure using McDonald’s Omega.

Finally, we conducted an exploratory analysis that was not pre-registered. Specifically, we assessed measurement invariance based on sexual identity (straight/ sexual minoritised). We tested three levels of measurement invariance (i.e., configural, metric, and scalar) by constructing each model and using the *anova* function to each model.

### Results

#### Preliminary Analyses

Before conducting the primary analysis, we assessed whether our data was appropriate for a CFA. We found no missing data in the HAS measure, meaning no imputation of missing values was required. Next, we assessed the normality of each of the 16 HAS items by ensuring they did not exceed ± 2. We found that all items had skew and kurtosis values within acceptable limits. We likewise assessed the skew and kurtosis for each of the measures within our analysis and found these within acceptable limits. As such, we proceeded with our analyses as planned.

#### Confirmatory Factor Analysis (CFA)

To test our first hypothesis, we conducted a CFA based on the factor structure in Table 2. The initial model showed acceptable fit: χ²(97) = 225.03, *p* < 0.001; CFI = 0.944; TLI = 0.933; RMSEA = 0.081 (90% CI: [0.067–0.095]); SRMR = 0.050. While CFI, TLI, and SRMR indicated a good fit, the RMSEA was slightly above the ideal threshold of 0.08, suggesting a moderate fit.

To address this, we examined modification indices, which indicated potential improvements by allowing certain items to covary. We permitted covariances between items 3 and 4, 6 and 9, 26 and 27, and 2 and 11, as these items had similar wording and tapped into closely related constructs. While theoretically justified, this adjustment yielded only a small change in the fit indices, suggesting that the slightly elevated RMSEA may be due to sample size sensitivity rather than substantive model misfit (χ²[97] = 225.03, *p* < 0.001; CFI = 0.944; TLI = 0.930; RMSEA = 0.081 (90% CI: [0.067–0.095]); SRMR = 0.050. Overall, the model provided an adequate fit, with the CFI, TLI, and SRMR supporting the model’s robustness despite the RMSEA being marginally elevated. We deemed the model suitable.

We next examined the factor loadings for each of the 16 items. As shown in Table 3, each item in the HAS loaded well onto its respective factors, with factor loadings ranging from 0.70 to 0.90. Notwithstanding the moderate RMSEA values, we found evidence to support our first hypothesis that the HAS would demonstrate good factor loadings and model fit.

**Table 3:** Factor loadings for the final 16-item HIV anxiety scale (HAS)

Item	Loading
*Factor 1: psychosocial implications of HIV*	
3. I worry that HIV will impact current or new intimate and romantic connections.	0.77
4. I worry about the impact of HIV on my sex life.	0.75
5. I worry about how living with HIV would affect my friendships.	0.83
6. I am afraid my family would treat me differently if I were living with HIV.	0.70
9. I fear people would judge me if I had HIV.	0.90
14. I worry about how living with HIV would affect how I see myself.	0.85
19. I worry that I would face discrimination at work if I had HIV.	0.77
20. I fear I wouldn’t have anyone to talk to if I had HIV.	0.72
*Factor 2: lifestyle implications of HIV*	
2. I worry about how living with HIV would affect my finances (i.e., money).	0.82
11. I worry that living with HIV will affect my career.	0.81
12. I worry about my ability to travel if I have HIV.	0.72
13. I worry that having HIV would affect my access to healthcare.	0.76
15. I fear having HIV would affect my access to housing.	0.71
*Factor 3: HIV testing anxiety*	
26. I’ve felt panicked when thinking about testing positive for HIV.	0.86
27. I’ve felt panicked when thinking about having an HIV test.	0.81
28. The idea of waiting for HIV test results makes me feel overwhelmed.	0.86

The final HAS, including all items and the scoring manual, can be found on our OSF. The measure is freely available to use under CC-BY-4.0 licensing. For the final measure, the items are numbered from 1 to 16 in the order they are presented in this table

#### Validity and Internal Consistency

Table 4 shows the correlations between each variable, the internal consistency of the measures and their confidence intervals.

***Convergent Validity.*** We first assessed the convergent validity of the HAS. As mentioned, we hypothesised that the HAS (and its subscales) would be positively correlated with health anxiety (measured using the S-HAI). We found support for this; the HAS total and each subscale scores were significantly correlated in the positive direction with health anxiety. However, we note that this correlation was weak.

We also hypothesised that the HAS (and its subscales) would significantly positively correlate with overall quality of life. We did not find support for this hypothesis. Neither overall HAS scores nor the subscales correlated significantly with a person’s quality of life. This is surprising given that quality of life was significantly associated with general health anxiety. We consider this more in our discussion section.

Finally, we also hypothesised that the HAS (and its subscales) would be significantly positively associated with the perceived risk of HIV (as measured by the PRHS). We found support for this, with the HAS and each subscale being significantly positively correlated (weak to moderate) with the PHRS. The HIV Testing Anxiety subscale had the strongest correlation, which makes theoretical sense considering the items in this subscale.

Taken together, our findings offer partial support for the convergent validity of the HAS. It may be that quality of life is a concept not related to HIV anxiety; greater HIV anxiety may not have a direct influence on a person’s quality of life.

***Divergent Validity.*** We next examined the divergent validity of the HAS by comparing scores on this measure with other measures that should be conceptually distinct (i.e., internet gaming disorder and social desirability). We found that total HAS scores were significantly correlated with IGDS (*r* = 0.15, *p* = 0.030). While significant, examining the lower confidence interval for this correlation shows it approaching zero. As such, this correlation should be interpreted cautiously. We found a similar significant correlation between the Lifestyle Implications of HIV subscale and IGDS (*r = 0.16*,* p* = 0.030). This association makes some theoretical sense, as participants who worry about HIV having an impact on their lives may extend this worry to their gaming habits. However, the lower confidence interval for this weak correlation is approaching zero and should be interpreted cautiously.

We also explored social desirability scores. We found that social desirability was negatively associated with both the Psychosocial (*r* = − 0.19, *p* = 0.01) and Lifestyle Implications of HIV subscales (*r* = − 0.15, *p* = 0.04). These findings suggest that individuals with higher tendencies toward socially desirable responding are more likely to underreport HIV-related anxiety in these specific domains. Participants who prioritise presenting themselves in a favourable light may minimise their anxiety in areas they perceive as socially sensitive or stigmatised, such as the potential lifestyle disruptions caused by HIV or the psychological toll of living with HIV anxiety. Despite these findings, the associations between the HAS and social desirability were weak.

Thus, we found partial support for the HAS measure’s divergent validity, both overall and regarding each subscale. Importantly, while we did find some associations contrary to our hypotheses, these significant associations were generally weak or, in the case of the IGDS associations, made theoretical sense. Given this, and combined with the stronger evidence for convergent validity, we deemed the HAS measure to show acceptable construct validity.

***Internal Consistency***. We also examined the internal consistency of the HAS measure by computing McDonald’s Omega (*ω).* As shown in Table 4, internal consistency was excellent for the overall HAS measure and each subscale. 

**Table 4:** McDonald’s Omega, and correlations between each variable with confidence intervals

Measure	McDonald’s ω	1	2	3	4	5	6	7	8	9
1. HAS total	0.89 [0.85, 0.91]	–	0.79***	0.91***	0.83***	− 0.10	0.26***	0.33***	0.15*	− 0.14
2. HAS—psychosocial	0.93 [0.91, 0.94]	0.74, 0.84	–	0.75***	0.62***	− 0.11	0.16*	0.29***	0.09	− 0.19*
3. HAS—lifestyle	0.88 [0.84, 0.90]	0.89, 0.93	0.69, 0.81	–	0.53***	− 0.07	0.23***	0.17*	0.16*	− 0.15*
4. HAS—testing	0.89 [0.85, 0.91]	0.78, 0.87	0.53, 0.70	0.43, 0.62	–	− 0.11	0.23***	0.46***	0.10	− 0.08
5. WHOQoL	0.87 [0.84, 0.89]	− 0.23, 0.04	− 0.24, 0.03	− 0.21, 0.07	− 0.24, 0.03	–	− 0.39***	− 0.12	− 0.22***	0.13
6. S-HAI	0.92 [0.89, 0.94]	0.12, 0.38	0.02, 0.29	0.09, 0.35	0.09, 0.35	− 0.50, − 0.27	–	0.23***	0.23***	− 0.11
7. PRHS	0.77 [0.72, 0.81]	0.20, 0.45	0.16, 0.41	0.03, 0.30	0.34, 0.56	− 0.26, 0.02	0.10, 0.36	–	0.24***	− 0.08
8. IGDS	0.92 [0.90, 0.94]	0.01, 0.28	− 0.05, 0.23	0.02, 0.29	− 0.04, 0.24	− 0.34, − 0.08	0.09, 0.35	0.10, 0.36	–	− 0.11
9. M-C-SDS	–	− 0.27, < 0.01	− 0.32, − 0.06	− 0.28, − 0.01	− 0.22, 0.06	− 0.01, 0.26	− 0.24, 0.03	− 0.22, 0.06	− 0.24, 0.03	–

Square brackets represent the 95% CI. Pearson’s *r* above the diagonal. 95% CIs below the diagonal

WHOQoL = Quality of Life, S-HAI = Health Anxiety, PRHS = Perceived Risk of HIV, IGDS = Internet Gaming Disorder, M-C-SDS = Social Desirability. Given the binary nature of the M-C-SDS, we do not compute internal consistency estimates for this measure

*p < 0.05, **p < 0.01, ***p < 0.001

#### Sexual Identity Measurement Invariance

We conducted an exploratory analysis of the HAS to assess its measurement invariance based on sexual identity. For simplicity, we assigned all participants who identified as a sexual identity (regardless of their gender identity) other than straight to a sexual minoritised identity (we excluded participants who indicated that they would prefer not to say and those who identified as demisexual). As such, we tested invariance variance across sexual identity (straight/sexual minoritised).

As shown in Table 5, the results demonstrate that the HAS measure exhibits an acceptable fit at all levels of invariance testing. Configural invariance indicates that the factor structure is equivalent across groups, supporting the use of the measure for both groups. Metric invariance further confirms that factor loadings are comparable, with no significant differences observed compared to the configural model. Lastly, scalar invariance shows an acceptable fit and no significant differences relative to the metric model, affirming that the HAS measure is suitable for assessing group differences across sexual identity.

**Table 5:** Sexual identity configural, metric and scalar invariance on the HAS

Model	df	AIC	BIC	χ2	RMSEA	*p*
Configural	194	8977.8	9338.4	326.58	–	–
Metric	207	8967.5	9285.5	342.25	0.05	0.267
Scalar	220	8950.2	9225.6	350.94	< 0.01	0.795

Significance test here refers to the chi-square difference test

Interestingly, all models tested during the measurement invariance stage evidenced excellent model fit (RMSEA < 0.08). This further confirms the robustness of the HAS.

## General Discussion

This study aimed to develop and validate a measure of general HIV anxiety using a general sample of HIV-negative (or status unknown) individuals from the UK. Before this research, existing measures of HIV anxiety were outdated and did not account for modern advancements in HIV treatment, testing, and prevention. Developing a new measure of HIV anxiety in the context of *U = U*, where prevention tools like PrEP are becoming more accessible, is crucial for understanding the nuances of HIV anxiety among HIV-negative individuals. However, given the current inequities in PrEP knowledge and access, it is crucial that the measure remains robust and applicable across diverse demographics [22]. This work promotes effective testing, treatment, and prevention strategies while assisting clinicians and researchers in identifying populations that may be most at risk for HIV anxiety and its associated healthcare implications. The current study developed and began the process of validating the 16-item HAS (HIV Anxiety Scale). Two focus groups with participants informed the final HAS, ensuring it is relevant to at-risk populations (i.e., men who have sex with men). The HAS assesses multiple dimensions of HIV anxiety: anxieties relating to (1) Psychosocial and (2) Lifestyle Implications of HIV and (3) HIV Testing Anxiety. The current study also supports the HAS as a reliable, valid, and robust measure of HIV anxiety, which will be useful in both healthcare and research settings.

Globally, there is an increasing focus on testing, treatment, and prevention of HIV to meet the UNAIDS target of eliminating new HIV transmissions by 2030 [2]. Regional governments, such as the UK, are also setting ambitious goals in line with this objective [46]. However, one significant barrier to achieving these targets is HIV anxiety, which can hinder individuals from accessing HIV testing, prevention, and subsequent treatment [16]. HIV anxiety may lead to avoidance of testing, potentially increasing transmission rates, or avoidance of HIV prevention information, which could elevate the risk of HIV for affected populations and others as well [4]. The HAS can be used to help identify these populations.

The HAS addresses the need for a psychometrically robust measure to assess HIV-related anxieties in a non-discriminatory manner. The measure has been guided by the principles of people-first language and directly informed by contributions from participants and field experts [47]. This provides a tool for future research that promotes health equity [48]. It has great potential for clinical application, especially in establishing PrEP eligibility and making sexual health counselling more patient-focused [49, 50]. By integrating HIV anxiety assessments into clinical practice, healthcare providers can better tailor interventions to individual needs, fostering a more supportive environment for patients. Regarding public health, a standardised measure can lead to establishing targeted interventions for reducing HIV anxiety and increasing testing and positive treatment outcomes [51]. For example, it can be used to inform evidence-based policy development that addresses the psychological burden of HIV anxiety and reduces barriers to testing and prevention methods.

Our findings show that neither the HAS nor its subscales correlated significantly with measures of quality of life (QoL), underscoring the distinct nature of HIV anxiety compared to general health anxieties– which is often associated with poorer QoL outcomes [52]. HIV anxiety is uniquely shaped by the profound stigma and social implications associated with the virus, framing it as both a social and an infectious disease [53]. Concerns about judgment, discrimination, and social rejection may isolate HIV anxiety from broader health anxieties, thereby exerting a more compartmentalised impact on QoL. The specificity of HIV anxiety often targets discrete life domains, such as sexual well-being, mood, and mental well-being. These focused concerns may limit its broader influence on overall QoL assessments.

Cognitive appraisals of HIV anxiety tend to centre on hypothetical scenarios or behaviours closely tied to risk management (e.g., undergoing HIV testing; [54]), which may further constrain its pervasive effect. In contrast, general health anxiety, with its non-specific nature, is more likely to impact multiple facets of life, resulting in greater disruption (e.g., sleep or body image; [55]). Coping mechanisms for HIV anxiety may also differ fundamentally. Psychological avoidance strategies [20] or a perceived sense of control over one’s risk of HIV [56] may help mitigate the impact of HIV anxiety more broadly on QoL. Conversely, general health anxiety often stems from fears of uncontrollable or unpredictable conditions, which can exacerbate its impact on QoL [57].

### Limitations and Direction for Future Research

Our research focused on developing and validating a measure of HIV anxiety. However, further research is necessary to confirm the usefulness of the HIV Anxiety Scale (HAS). We recommend that future studies investigate the HAS’s factor structure and its validity across various samples, cultures, and settings. For instance, future research may wish to validate the HAS in a clinical sample and the predictive validity of the measure in a healthcare setting (i.e., do measures on the HAS predict future testing behaviours). Similarly, future research could benefit from assessing the test re-test reliability of the measure. As we mentioned above, certain groups, such as men who have sex with men, are at greater risk of HIV transmission. Future research should ensure this general measure is valid with those at-risk populations, given the utility of this measure might be more felt within these contexts.

Owing to the discrete differences between HIV anxiety and general health anxiety, future research may also benefit from investigating specific mediators, such as perceived social support, to delineate the mechanisms by which HIV anxiety and general health anxiety exert differential effects - especially on QoL outcomes. These insights could inform tailored interventions to address the unique psychological and social burdens associated with HIV anxiety. We only developed the HAS in English, so future research may wish to adapt the measure in different languages. We encourage the HAS to be used internationally and in diverse healthcare settings. However, we acknowledge that adaptions to the HAS may be needed to assess culture/region-specific (e.g., specific healthcare pathways for certain regions) predictors of HIV anxiety.

A specific limitation of the HAS is the significant (albeit weak) associations between the Psychosocial and Lifestyle Implications subscales and social desirability. Specifically, the influence of social desirability on certain subscales indicates that self-reported HIV anxiety scores may be somewhat suppressed in individuals who aim to present themselves in a socially favourable manner. This bias may limit the sensitivity of the Psychosocial and Lifestyle subscales in capturing the full extent of HIV anxiety in these domains. However, we note that these associations were weak.

## Conclusion

The HIV Anxiety Scale (HAS) is a validated tool for measuring HIV-related anxiety in healthcare and research. Although validated in a UK sample, its design may apply cross-culturally in other Western countries with similar HIV dynamics. Developed with a people-first language, the HAS aligns with UNAIDS’ 2030 goal of ending HIV transmission. It offers a standardised tool to inform targeted interventions for HIV testing, prevention, and treatment. The measure encourages future research, addresses psychological aspects of HIV care, and promotes a holistic approach to prevention and treatment.

## Data Availability

The data and R script supporting our study’s findings are openly available on the Open Science Framework (OSF) at https://osf.io/uwspe.
